# Application of Modern Multi-Sensor Holter in Diagnosis and Treatment

**DOI:** 10.3390/s20092663

**Published:** 2020-05-07

**Authors:** Erik Vavrinsky, Jan Subjak, Martin Donoval, Alexandra Wagner, Tomas Zavodnik, Helena Svobodova

**Affiliations:** 1 Institute of Electronics and Photonics, Faculty of Electrical Engineering and Information Technology, Slovak University of Technology, Ilkovicova 3, 81219 Bratislava, Slovakia; jan.subjak@stuba.sk (J.S.); martin.donoval@stuba.sk (M.D.); tomas.zavodnik@stuba.sk (T.Z.); 2 Institute of Medical Physics, Biophysics, Informatics and Telemedicine, Faculty of Medicine, Comenius University, Sasinkova 2, 81272 Bratislava, Slovakia; 3 Department of Simulation and Virtual Medical Education, Faculty of Medicine, Comenius University, Sasinkova 4, 81272 Bratislava, Slovakia; alexandra.wagnerova@fmed.uniba.sk (A.W.); helena.svobodova@fmed.uniba.sk (H.S.)

**Keywords:** multi-sensors, Holter, electrocardiography, electromyography, electrodermal activity, inertial measurement unit, pulse-oximetry

## Abstract

Modern Holter devices are very trendy tools used in medicine, research, or sport. They monitor a variety of human physiological or pathophysiological signals. Nowadays, Holter devices have been developing very fast. New innovative products come to the market every day. They have become smaller, smarter, cheaper, have ultra-low power consumption, do not limit everyday life, and allow comfortable measurements of humans to be accomplished in a familiar and natural environment, without extreme fear from doctors. People can be informed about their health and 24/7 monitoring can sometimes easily detect specific diseases, which are normally passed during routine ambulance operation. However, there is a problem with the reliability, quality, and quantity of the collected data. In normal life, there may be a loss of signal recording, abnormal growth of artifacts, etc. At this point, there is a need for multiple sensors capturing single variables in parallel by different sensing methods to complement these methods and diminish the level of artifacts. We can also sense multiple different signals that are complementary and give us a coherent picture. In this article, we describe actual interesting multi-sensor principles on the grounds of our own long-year experiences and many experiments.

## 1. Introduction

The human body is a good conductor, allowing easy recording of electrical signals generated by the human body. That fact was applied by William Einthoven when he measured electric potentials of the heart at the beginning of the 20th century. An easier way to measure the heart electric activity was shown by Norman Jeffrey Holter in the second half of the 20th century. He started his trials with the long-term recording of human physiological parameters on active people [1]. He fixed his experiment on portable devices for recording heart activity–electrocardiographic (ECG) Holter [2]. The first Holters were large, uncomfortable, and required a chest lead unit, including a radio broadcasting unit with an antenna system [3]. Thanks to technical progress, nowadays, Holters are small portable devices [4] with wireless signal transmission, integrated memory for data recording, and modern electrodes. They are used for patient monitoring in the comfort of their own home where they are not scared by doctors. The so-called white coat syndrome has disappeared.

Holters are great for intercepting different heart or breath abnormalities [4,5,6,7,8,9,10], fetal arrhythmias [11], or simple physiological human states [12,13,14,15]. Holters showing daily ECG records are the perfect tools for long-term monitoring of the patient’s physiological state, able to notice deviations, which are not frequently repeated. Many patients often come to the ambulance saying they were sick the day before but feel fine at present, with ECG records showing no anomalies. This frequently happens in the case of cardiac fibrillation, which can eventually create cerebral defeat. Therefore, a classic ECG, which is just like a short flash in a patient’s history, is insufficient.

With the advent of telecommunication and internet technology, medicine could expand its borders, creating a new field called telemedicine. First medical data were communicated by telegraph, and people and doctors later started to use telephone and, in the 20th century, internet [16]. For today’s communication and data transfer, low-power wide-area networks (LPWAN) are very promising [17,18,19,20]. Telemedicine is a very fast evolving and transforming sphere where Holter devices are used in home care or for monitoring human physiological parameters by mobile healthcare assistants [5,21,22,23]. Human health is very important and early accurate diagnosis is essential to sustaining a high quality of life. The hectic lifestyle of today’s world causes people to live in stress without enough rest, undermining their self-care of health. Hence, enhancing and inventing new diagnostic methods, which record human physiology and show diseases in the early stages, are necessary for the development of medicine and more effective treatments for patients at long distance. Innovative methods could inform people about their own physiological state nonstop, and if some health problems arise, doctors would be directly informed of the person’s health state. Data measured by Holters sent immediately to the doctor would allow the doctor to control their patient from different places [24,25]. In addition, in the case of health problems, the doctor could instantly consult the health state with a specialist, or act fast and send an ambulance, if necessary. In addition, telemedicine helps to disburden doctors from little work, as the market is literally overwhelmed by numerous new medical products aimed at everyday people. The conditions for data recording, however, can vary in different environments, and are not comparable to predefined hospital conditions, and the records of the signals suffer from lower quality. These disadvantages could be removed by utilization of advanced methods and the newest technical devices implementing integrated chips together with the increasing computing power of new microprocessors, and creating cutting-edge integrated medical devices. In the near future, we can expect various new modern integrated medical devices for home examination and medicine self-diagnostics considering the amounts of capital invested in this field.

In this article, we would like to summarize the actual state in modern Holters and describe the perspective and innovative sensing principles usable in multi-sensor monitoring. Likewise, we would like to outline short sections of selected experiments we accomplished using our own designed devices during a longer period of time. These experiments aim to widen the knowledge in the multisensory field.

## 2. Capability of Modern Multi-Sensor Holters

The advantages of multi-sensors and multi-channel sensing are in the possibility to measure the same variable to achieve greater reliability in a more difficult environment. For example, it is possible to measure the heart rate (HR) electrically from an ECG [26,27,28,29,30,31], optically using pulse-oximetry [26,32,33,34], mechanically by vibration from seismocardiography (SCG) [7,30,34,35,36,37,38,39], or from minor variations in electrodermal activity (EDA) [40,41].

### 2.1. Electrocardiography and Respiration

Most famous and historically, the ECG Holter is one of the first introduced wearable monitoring devices. Today, the market offers single-channel or multi-channel Holters, with many complementary sensors, with or without a hospital certificate. Their price varies from tens of euros [42] to several thousands (EC-12H 12-Channel Holter ECG system by LabTech, Hungary or CardioMera ECG Holter Monitor, Medusoft, Australia) [43,44,45,46,47] depending on the quality and properties offered.

However, there are still challenges in this area. When considering a simple ECG record, with the doctor, the patient is in a quiet environment and is supervised, the quality of the electrode contacts is verified, the patient is in the prescribed position (mostly lying), and, for a brief moment of signal recording, they usually hold their breath to reduce artifacts, and the doctor, if necessary, receives immediate patient feedback. On the other hand, in real-life ECG recording using a standard Holter, the doctor is not able to control the quality of the skin–electrode contact or the detailed position of electrodes if they are not wrongly set. He/she is not familiar with the patient’s physical or mental activity and, thus, cannot evaluate if the increased heart pulse is objective instead of tachycardia. Moreover, human posture (patient bent in an unnatural position or traveling in a car and the fluctuating ECG amplitude is related to vehicle shaking), as well as environment (increased breathlessness may be caused by elevated temperature and humidity), have to be taken in account. Therefore, the ECG recording in the home environment has to be supplemented by the recording of physical parameters of the environment, and it is necessary to correlate all these effects to the final evaluation. This requires increased work activity from the doctor, and it is necessary to extend his skills in analyzing such modified ECGs. Hence, the development of auxiliary software, diagnostic applications, implementation of databases, and neural networks, which simplify this specific and more complicated diagnostics, must go hand in hand [48,49]. Long-term monitoring also produces an enormous quantity of data, which requires efficient data coding and communication of measured signals to the remote clinical back-end systems, and therefore, several modern communication protocols like Constrained Application Protocol (CoAP), Message Queuing Telemetry Transport (MQTT), Message Queuing Telemetry Transport for Sensor Networks (MQTT-SN), and Advanced Message Queuing Protocol (AMQP) have been introduced [50], and there is an urgent need for evolution in automatized screening [51,52,53].

**Figure 1:**
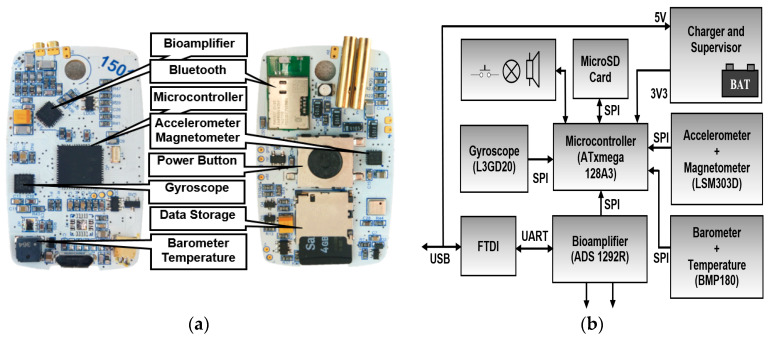
Electrocardiographic (ECG) Holter: (**a**) Printed circuit board (PCB) layout, (**b**) block diagram.

For research purposes, our laboratory has developed an ECG Holter platform (Figure 1) [54]. In order to incorporate a wide range of different scientific tasks, the Holter was designed to be as versatile as possible. The heart of this platform is based on an analog front-end ADS1292R (Texas Instruments, Texas, USA) and ATxmega 128A3 (Microchip Technology, Arizona, USA) microcontroller. The analog front-end also includes circuits for impedance respiration measurement. The overall device battery consumption starts at 3 mA, which is required for portable electronics. The ECG Holter is complemented by inertial measurement units (IMU): Accelerometer with magnetometer LSM303D STMicroelectronics, gyroscope L3GD20 STMicroelectronics, barometer with temperature sensor BMP180 Bosh and notch filter. The gain, range, and sample frequencies for all units can be set in a wide range using a configuration file or Bluetooth control. Data are stored to a built-in 16 GB SD card in Comma-separated values (CSV) format, with the possibility of conversion to European Data Format Plus (EDF+) format. Measured data can be transferred offline via a USB connector or online via a Bluetooth Low Energy 4.0 (BLE) interface.

**Figure 2:**
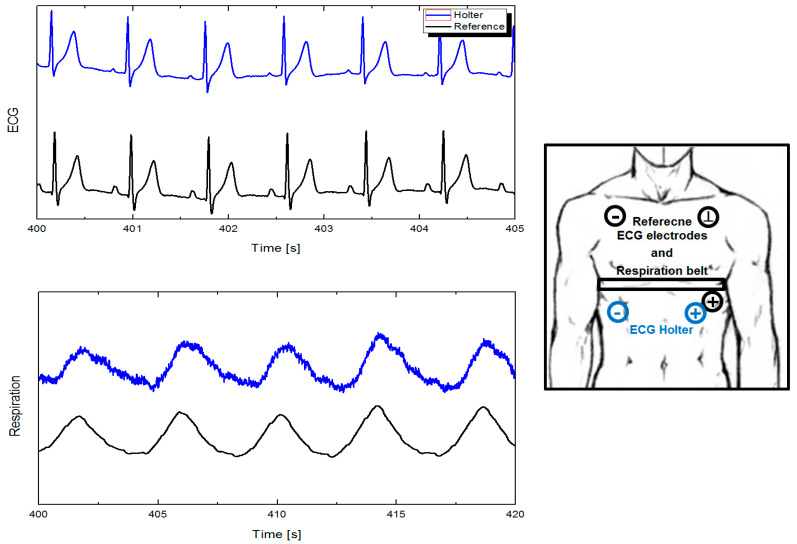
Comparison of ECG and respiration achieved by laboratory instruments and Holter.

In Figure 2, we performed a short validation measurement where we compared our ECG Holter with a laboratory instrument. The aim was to verify the detailed parameters of the used Holter, including how the signal is affected when different electrode placing is used, if commercially used automated software correctly evaluates our output signals, and, of course, the progress in the process of hospital certification. We were also interested in the overall behavior and reliability of the innovative impedance respiration sensing [54], how it will stand compared to indirect measurement through pressure sensors, and the resistance of the circuit rib cage - chest belts [55,56,57] in different life situations. The ECG Holter signal was post-processed using a digital band-pass filter in range of 1–100 Hz with an auto-adjustable transition width. The respiration was filtered using the same filter in the range of 0.05–3 Hz. We observed that the results were comparable, and even in some situations such as low physical load or conversation, the impedance monitoring performed better. This can also be supported by the fact that the respiration impedance sensing technique has started to be widely used today [58,59,60].

### 2.2. Inertial Measurement Units and Seismocardiography

Inertial measurement units (IMU) are mostly used to record the movement, position, and posture of the human body [61,62,63,64,65,66] or for classification of human daily activities [67]. The first versions were used as simple pedometers, but at present, the range of applications is fast expanding. The main reason is their very low cost and easy implementation. Currently, they are inserted into almost all wearable instruments [7,36,68] and can also be used for indoor navigation.

In today’s highly industrialized time, the overall physical activity of the population falls below the recommended levels. As a result, obesity and diabetes are spreading all over the world. Obesity is often labelled as the epidemic of the 21st century. Monitoring childhood obesity, where accelerometers offer an objective measure of habitual activity independent of self-reporting [69,70], or homecare monitoring of elderly persons, where the system records their activities, events, and potentially important medical symptoms [71,72], is very well known.

As mentioned earlier, it is very important to correlate the ECG, heart rate variability (HRV) signal, and physical activity (IMU). This minimizes the number of false alarms and gives a more complete overview of the ECG record and its abnormalities.

The next application that seems to be interesting is seismocardiography (SCG). SCG measures small thorax movement and vibrations that contain information related to the cardiovascular and respiratory system [7,35,36,37,38]. The thorax accelerometer signal contains a low-frequency component corresponding to the motion of the chest wall due to respiration and a higher-frequency component corresponding to the heartbeat. Actual research teams are either trying to develop extracting algorithms of these signals [73] or are focused on increasing the quality of daily-life ECG monitoring using quantitative analysis of motion artifacts [74,75]. In different studies, the quality of the obtained SCG is enhanced using a novel adaptive recursive least-squares filter [76], time–frequency distribution analysis [77], or even using two cooperating accelerometers [78]. Very interesting are the studies where relations between ECG and SCG waveforms are analyzed [79], and then the simultaneous acquisition of ECG and SCG signals followed by mechanisms for the automatic delineation of relevant feature points can distinguish between normal and abnormal morphology [80], detect critical cardiac behaviors, and build early warning systems [81].

**Figure 3:**
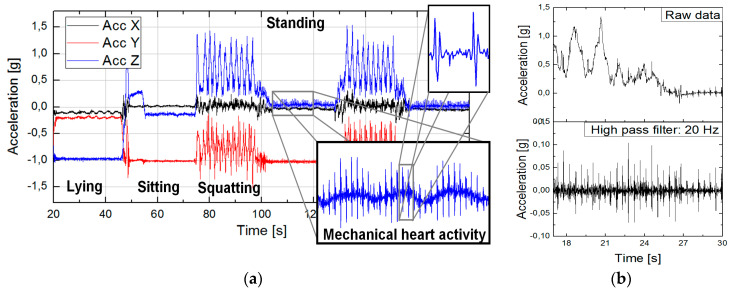
Holter inertial measurement units (IMU): (**a**) Body movement and seismocardiography (SCG), (**b**) heart rate (HR) during high physical activity.

Figure 3a shows an example of signals obtained using our Holter with its IMU fixed to the thorax [68]. Except the overall movement and position of a human, a detailed look of the heart and respiration activity (mechanical heart activity) can be captured. Using a simple 20 Hz high-pass filtering, HR can be obtained even during demanding exercise when the ECG signal is unreadable due to heart bouncing (Figure 3b). Detailed accelerometer signals can be used, e.g., for cough and swallow analysis [82].

### 2.3. Muscle Activity

Electromyography (EMG) is used in motion analysis, physiotherapy, clinical research, and sport training [83,84,85,86,87,88]. EMG signals can often be used in automation for prosthetic devices such as prosthetic hands or lower limbs [89]. However, sensing in this area has certain specifics. Myoelectric prostheses expect comfortable and reliable electrodes without interfering with the user’s daily life. They must be ventilated, flexible, and foldable. Polymers, like polysiloxane [90], conductive fabric [91], or textile electrodes made by screen printing technology [92] meet the requirements.

In measuring muscle activity, the application of accelerometers seems to be very interesting, and the relevant field of science is called mechanomyography (MMG) [93]. MMG refers to the surface measuring of small vibrations of loaded muscles (amyostasia) or even a single motor unit [94]. Although there exist alternative methods of sensing muscle vibration, like piezoelectric resonance-based sensors [95] or laser Doppler [96], accelerometer sensing of MMG is still the most common. MMG can be a useful alternative to the electromyogram (EMG). It has a higher signal-to-noise ratio (SNR) than surface EMG and it can monitor the activity of even deeper muscles. This technique is, therefore, often utilized for evaluation of the muscular fatigue [97,98,99,100,101] or of the mechanical delay of muscle contraction [102,103]. The combination of MMG and EMG was already investigated for rehabilitation, control of prostheses, and in robotics. If MMG is added as a second detector to EMG monitoring, the total error of devices and prostheses can decrease up to 50% [89].

Electrical impedance myography (EIM), which measures the impedance of the electrical potential generated by muscles and neuron cells, can also be classified among innovative myographic methods. It can be used for observing muscle health and conditions. For example, there is a report for usage in the diagnosis of neuromuscular disorders [104], or for capturing changes in muscle composition [105]. In the study by Ma et al. [106], a wearable motion capture and measurement system combining an EMG, MMG, and ultrasound probe for understanding locomotion was developed.

**Figure 4:**
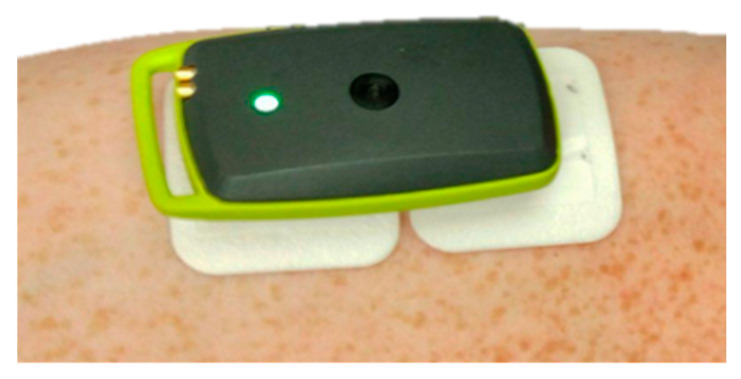
Electromyographic (EMG) Holter fixed to the body using Ag/AgCl electrodes.

**Figure 5:**
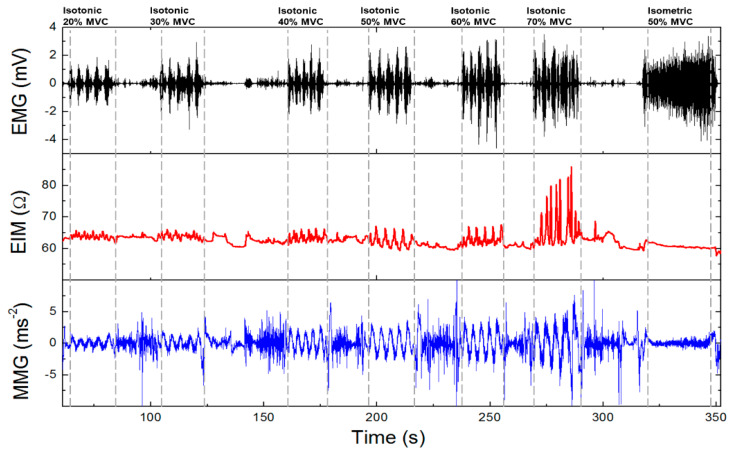
Electromyography (EMG)/electrical impedance myography (EIM)/mechanomyography (MMG) signal of one volunteer.

**Figure 6:**
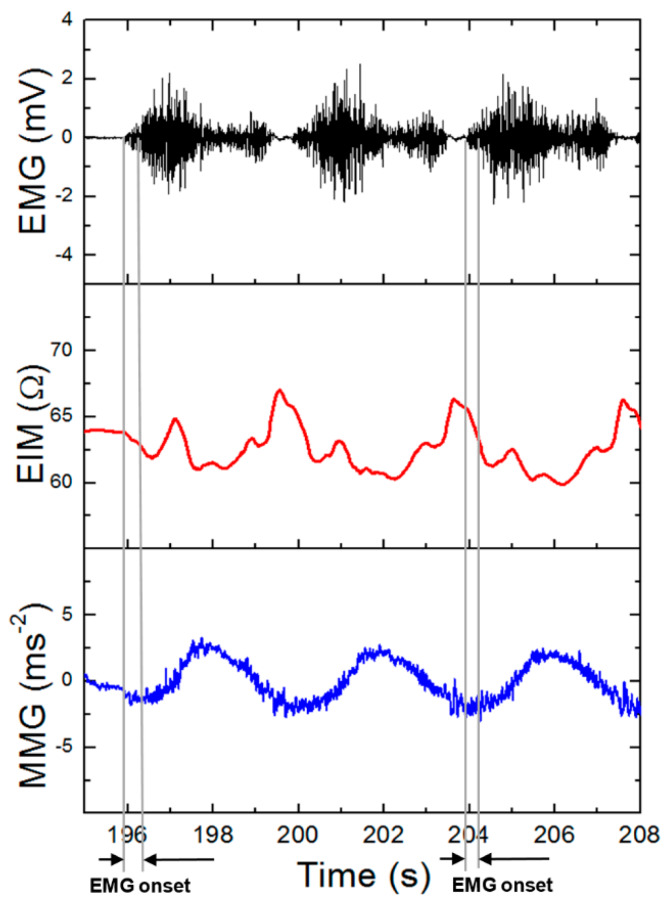
Detailed signal with transition of isotonic movement to isometric.

**Figure 7:**
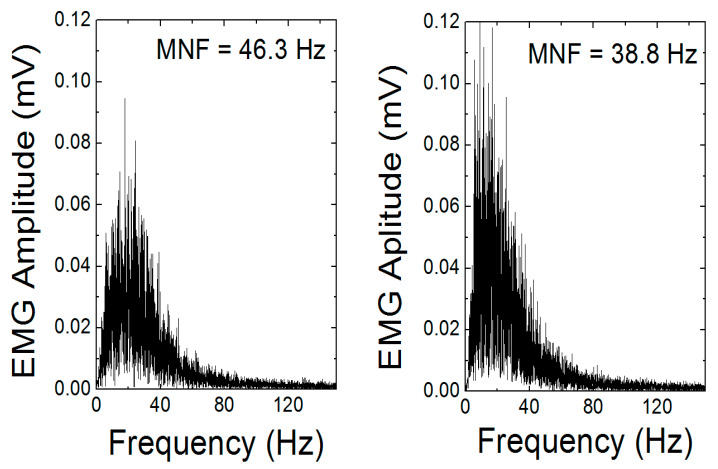
Power signal density. Amplitude and mean frequency mean frequency (MNF) shift of power signal density (PSD) due to fatigue.

When looking at our results, we demonstrated the capability of the combination of EMG (electromyography), MMG (mechanomyography), and EIM (electrical impedance myography) for examining muscle activity [107]. These variables can be measured simultaneously only by redesigning the electrode contacts and software setting of previously introduced Holter parameters. Instead of using cable contacts, we mounted the Ag/AgCl electrodes with clips directly to the bottom part of the housing in a distance of 2 cm (Figure 4). Compared to ECG measurements, we increased the sampling frequency of biopotential and impedance measurements from 1000 to 2000 Hz and for vibration measurement from 100 to 400 Hz. When measuring vibrations, the range was also increased from ±2 to ±4 g. Obtained signals were again software-filtered. The EMG signal used a digital band-pass filter in the range of 1–500 Hz, EIM, and MMG using a 0.1 Hz high-pass filter. Measurement was performed on the biceps brachii muscle. In Figure 5, the total time response of all signals is presented. The exercise comprised six series (each five lifts) of gradually increasing isotonic load (20–70% maximum voluntary contractions (MVC)), followed by 30 s of isometric exercise with 50% MVC (maximum voluntary contractions). Detailed analysis (Figure 6) shows that there is visible delay between the muscle activation (EMG start) and movement (MMG shift). These phases represent the transition of isotonic movement to isometric. This delay is interesting from the perspective of the evaluation of reflexes and reactions. For robotic and prostheses control, it is helpful that EIM is sensitive only in the case of isotonic signals and not isometric signals (Figure 5 and Figure 6). Figure 7 interprets the typical power signal density (PSD) of an isometric load. The comparison shows the amplitude and mean frequency (MNF) shift due to the fatigue factor.

The capability of muscle activity Holters can grow rapidly when connected together in a synchronous network, demonstrated, for example, in the case of EMG Holters by Delsys Incorporated (MA, USA) in their Trigno platform or by BTS Bioengineering Corp. (MA, USA) in their FREEEMG series [108,109,110,111,112]. As interesting research study for the precise control of prosthetic devices, the surface potential mapping [113] or stretchable EMG patch sensor integrated with the miniaturized wireless system modules, should also be mentioned [90].

### 2.4. Electrodermal Activity (EDA)

Further, there is growing interest and promise for wearable devices for electrodermal activity (EDA) recording, often also known as the electrodermal response (EDR) or psychogalvanic reflex (PGR) [114]. Laboratory devices commonly measure electrodermal activity between the down part of two fingers of the non-dominant hand. Such devices are ideal for psychological research, lie and stress detectors, etc. For daily life, more suitable EDA meters are in the form of watches, wristbands [115,116,117,118], or even smart eyewear [119] and bras [120], which are more practical and comfortable. The imperfection is in decreased sensitivity, while the nervous reactions and change in skin conductivity are more significant in the palm areas, where the stratum lucidum and potential barrier are presented [121,122,123]. Another problem is that the signal can be, due to a high movement artifact, obtained only in the calm state, so again, cooperation with IMU or at least the accelerometer is required.

**Figure 8:**
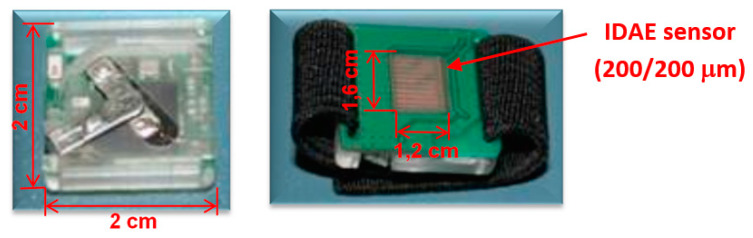
Electrodermal activity (EDA) Holter.

**Figure 9:**
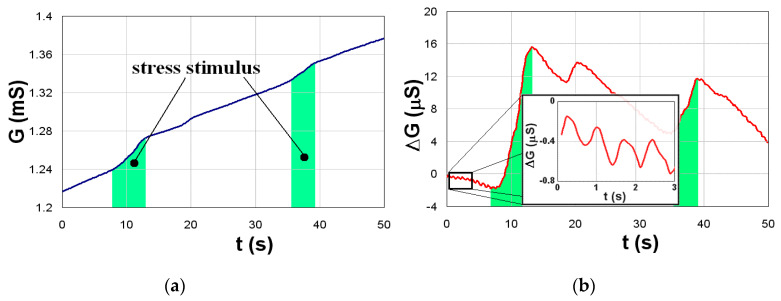
Typical time responses of EDA: (**a**) Original signal, (**b**) filtered signal with visible heart-pulses.

We tried to avoid these problems by constructing an EDA Holter in the form of a practical ring [124] (Figure 8), which does not sense the EDA between the fingers like a conventional device, but locally on a small place in an area of 1 × 1 cm (Figure 9a). Such an EDA ring is more suitable for daily wear and the signal artifacts are lower. The Holter generator generates a sinus signal with an amplitude of 1.6–3 V and frequency of 1 kHz to a gold-plated interdigitated array of electrodes (IDAE). Their size is 200/200 m, so the electric field enters only into the neuroactive areas (stratum lucidum–potential barrier) and the impedance changes are quicker and more bound to the psychogalvanic response. Total dimensions of the EDA Holter are 20 × 20 × 5 mm and the device is connected to a smartphone with Bluetooth 2.0. The data are stored in CSV format. To minimize polarization effects in the skin, also called electrodermal phenomena, we used software filtering of this drift based on periodical recalibration with an exponential function. An exponential approximation was performed every minute, and this exponential was subtracted from the measured data (Figure 9b). This experiment also led to a very important result: The microelectrode probes are able to monitor the electrodermal response, as well as the heart pulses, simultaneously. They are present as small variations in the EDA signal related to blood pulsating in the bloodstream–plethysmography.

### 2.5. Pulse-Oximetry–Photoplethysmogram

The next sensor principle we describe in this article is pulse-oximetric, which is closely related to the photoplethysmogram (PPG). Classical sensors are commonly used in hospitals to monitor heart rate (HR) and blood oxygen level (spO_2_) mostly using the transmittance principle on fingers or earlobes [125,126,127], but there are also less traditional variants like PPG sensors placed in the human trachea during anesthesia [128]. In homecare, they are usually known to be implemented in all types of smart watches [129,130,131,132,133], and they work on a little more complicated but suitable reflectance principle for nonstop wearing [134,135,136,137]. They use mostly red, infrared, or green light to monitor HR, and their combination is used to determine spO_2_ levels. In fact, this method is arguably the most used for measuring HR. To remove false values and increase reliability, PPGs are sometimes enhanced by accelerometers [138] or multiplied [139,140] so they can be deployed for detailed heart rate variability (HRV) analysis [141], fibrillation classification [142], arterial status (ageing) monitoring [140], or in demanding applications like automotive applications where the PPG signal is measured from the palm of the hands [143].

**Figure 10:**
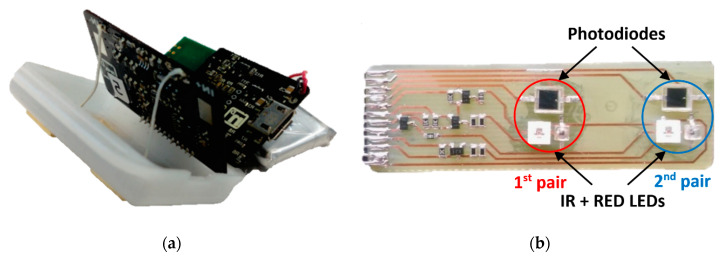
(**a**) Pulse-oximetry Holter, (**b**) paired pulse-oximetry sensors.

Our team have developed a pulse-oximetric Holter (Figure 10a), based on the reflective principle, and enhanced it using two synchronized pulse-oximeters at a predetermined distance of 2 cm, so it is possible to also measure the local blood flow rate. Our measuring system (Figure 10b) consists of two pairs of light-emitting diodes (LED). The used RED LED is a Vishay VLMR51Z1AA and IR LED VSMY2943RG. The wavelengths of the LEDs are chosen matching the optical properties of blood. Oxygenated hemoglobin has a maximum light absorption of 940 nm and, on the other side, reduced hemoglobin has this maximum of 660 nm. Thanks to this difference, we are able to determine the ratio of oxygenated and reduced hemoglobin. This ratio determines the level of spO_2_ directly. Both pairs of LEDs are coupled with Vishay BWP34 photodiodes, which have comparable sensitivity to both of the used wavelengths of the emitted lights. The signal from the photodiodes is hardware-filtered using a band-pass filter of 0.5–5 Hz. All of the measurement processes are controlled by a Holter, which is based on a JN5148 microcontroller with an integrated ZigBee communication module and on a 24-bit analog-to-digital converter with software-adjustable sample rate and gain. PC communication is provided by one receiver module (USB dongle), which can simultaneously communicate with 4 to 6 of such Holters [144].

In the last years, a huge effort has been made to use PPG for cuffless blood pressure (BP) determination. There are known algorithms where systolic and diastolic BP are calculated from the shape of the PPG curve [145]. Thanks to modern neural learning methods [146,147], the accuracy of the results is continuously increasing [148,149,150].

For accurate estimation of BP, the combination of PPG with other physiological parameters such as ECG, SCG, ballistocardiogram (BCG), impedance cardiogram (ICG), etc. seems to be very promising [151]. Blood flow rate partially correlates with blood pressure [152,153]. In practice, plenty of devices are now coming that measure the approximate blood pressure by the phase shift between the ECG and the PPG curve [154,155,156,157]. The principle is simple. It is assumed that the ECG signal spreads across the body at the speed of light, so it is recorded immediately, and the PPG signal reacts to the blood flow itself, making it “a little” slower. These devices are mostly in the form of smart watches, where there is an optical PPG sensor with one ECG electrode (one bottom electrode can also be added for noise reduction) on the bottom, and a second ECG electrode on the top of watches. Such a smart watch on the market, for example, is Glutrac, where they even try to determine the blood glucose from an advanced PPG [158]. A sensing method like this, of course, needs planned human cooperation, as they must hold the top electrode with their second hand in the I bipolar Einthoven lead for about 10–60 s. The devices fusing PPG and ECG with deep neural networks seem to be very promising for the future, combining together PPG shape recognition and PTT to increase the accuracy of results [159,160]. What is also interesting seems to be a device combining an ECG, PPG, and SCG placed on the human chest [161]. In this principle, we estimate that the blood pressure resolution will be decreased by the low separation distance between the PPG sensing location and the heart. Smartwatch CareUp [162] uses two pulse oximeters for obtaining BP: One placed on the back and the second on the front of the watch. BP is calculated from the time delay between them. The Omron company is trying to miniaturize the classical blood pressure cuff into smart watch bands [163].

**Figure 11:**
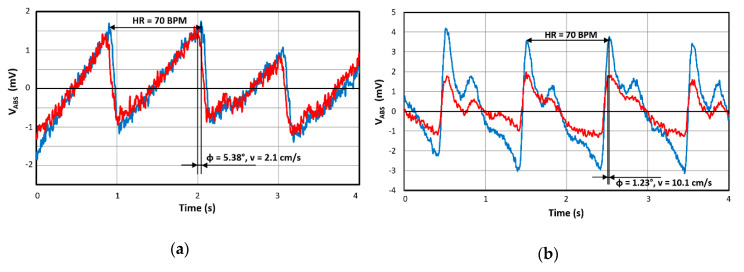
Signals from paired photoplethysmogram (PPG) sensors measured at: (**a**) Middle finger, (**b**) wrist.

However, returning back to our device that uses only an optical PPG principle, it has to be clarified why it is relevant to continue in this sensing principle. Our main goal is to synchronize ECG Holters on the chest with a smart watch enhanced by our double PPG sensor. Thanks to this concept, we will obtain not only the overall blood speed but also the local speed and, using the appropriate algorithm, we can suppress artifacts like vascular elasticity, hydration, drug effects, etc. and increase accuracy. Figure 11 shows a part of our results, where we see shifted PPG signals (output signals of photodiode, only RED diode active) from the middle finger and wrist, which corresponds to blood speeds of 2.1 and 10.1 cm/s. The heart rate was about 70 BPM in both cases.

### 2.6. Technolgies of Wearable Devices and Smart Clothing

All wearable technologies have a few common pitfalls that they must improve. These include battery life, miniaturization associated with non-invasiveness, and, last but not least, price. Cost-effectiveness is one of the most important goals for the adoption and implementation of wearable technologies. The more sophisticated the system, the higher their price, and therefore, only if they are available and reasonably priced, they can really have the potential to improve health care. However, the smaller the device developed while maintaining the functionality, the greater the price, and the same applies to batteries. The biggest problem is that there is always a demand for longer-lasting but smaller batteries at the same time. It seems that the solution is not in the batteries themselves but in various settings and optimizations of data transfer, components, or software for the best possible battery management. In the coming years, batteries may have twice the density, but due to the chemical laws of energy storage, they have no potential to improve over time, as we can see from semiconductors. The solution to this key problem of wearable sensor devices is, therefore, in the development and selection of the right sensors and components with low consumption, as well as the use of low-energy technologies for data transmission such as the LoRa, Sigfox, or NB-IOT network. It is these networks that have low bandwidth and can transmit data over long distances. There exist many studies and reviews about smart wearables, and recent advances and future challenges [164] about the creation of cooperative systems based on wearable devices are directed to the field research context [165] or the energy challenges for wearable sensing technologies [166]. In addition, studies about battery-free wearables are very usable, as demonstrated by Orfanidis et al. [167], where the authors demonstrated how LPWAN wearables can operate by using energy-harvesting, which illustrates that the LoRa radio is able to operate by a combination of solar and mechanical energy on a smart shoe prototype outdoors.

The current trend is the connection of Holters with clothing containing conductive or piezoelectric textiles and threads. All this can be categorized as smart clothes. New Holters can be worn on the body in a variety of ways such as a chest strap or belt. As they may be visible under light clothing or under shirts with a low neckline, current researchers, including our team, are trying to create and implement invisible Holters directly into smart clothes for unlimited wearing [168,169]. The market already provides several examples. The sports bra from Movesense contains textile electrodes and a detachable Holter containing HR, IMU, and Bluetooth [170]. Alternatively, the MyHeart Instrumented Shirt [171] is equipped with sensors from conducting and piezo-resistive materials on a textile structure integrated in fabric, which is able to monitor ECG, on-arm EMG, respiration rate, skin temperature, and body movements. Blood pressure and oxygen saturation can also be obtained on demand. Some research institutes are trying to measure body composition and hydration. In this research, a self-powered smart patch for sweat conductivity monitoring [172] or this wearable potentiostat [173] can also be helpful. Interesting also is the ECG-derived respiration, which is a method for determining the respiratory information from ECG. In our research [174], we optimized it for miniaturized Holters with a low inter-electrode separation distance (few centimeters), often available in the form of patches [75,175,176]. We found that for measuring ECG and derived respiration, it is best to use the position under the pectoral muscle and, if possible, with electrodes orientated parallel to the heart axis. The suggested electrode setup is suitable for common daily activities, if no high physical activity is present. The possibilities seem almost unlimited.

All new technologies also bring various challenges with them and problematic features that need to be addressed or improved. The basic problem of all sensor systems recording physiological data of the human body is the privacy and security of data. Wearable devices thus record, process, and store sensitive user data. Users are generally unaware of the real risks of losing privacy and often underestimate this form of security. The basic form of increased security is data encryption during recording and storage in the internal memory of measuring devices. Encryption is usually integrated in the processor/on the chip, what most of the reliable developers of such systems currently do. For example, the choice of on-chip encryption can affect processor selection and, thus, battery consumption. After measurement and storage, the encrypted data are moved, e.g., to a server where other protection elements can be used [177,178,179].

An inseparable part of the research of portable devices is also the connection of their production with the selection of materials and technologies so that they are practical, cleanable, and fashionable [180].

## 3. Conclusions

Nowadays, due to huge technical developments, telemedicine applications have ascended. New sensors, software diagnostics, database systems, and innovative sensing principles have been developing fast. Development of information technologies gives us a great opportunity to improve health systems and facilitate doctor’s work. 

Another advantage lies in the measurement of human body activity in home conditions, where people are calmer, more at rest, and not frightened by doctors. In these conditions, the results could be more appropriate. In addition, many people can suffer from diseases, when their heart demonstrates irregular activities often unnoticeable during ECG measurement in the ambulance; therefore, longer 24/7 recording with a Holter device is needed to catch the discrepancies.

Currently, in our laboratory, we are trying to apply these findings to modern smart clothes. We place the Holter on the chest, sew a suitably positioned electrode (using an optimized position of the electrodes for ECG recording), and place the improved pulse oximeter in the wrist or shoulder bracelet. If this is all combined, we could ultimately create a modern and robust device for a comprehensive record of human physiology. An integrated part of our research is the development of applications, access points, screening, diagnostic algorithms, and development of neural networks, in the form of powerful computers, as well as integrated directly inside Holters.

Taken together, in this work, we outlined some substantial options. Yet, there is a huge variety of other methodologies and applications, and their number will continue to grow with time. Likewise, Holter principles will be implanted in further devices of everyday use (e.g., our e-health mouse patent). We believe that we have shown a very broad application of multi-sensor Holters and that they have a bright future in diagnosis and treatment. We used our Holters introduced here, for example, in psychological research, for cognitive monitoring of the relationship of anxiety and allergies, in the investigation of ADHD attention in young children, in monitoring the impact of the working environment on performance, in apnea research, in determining the anaerobic threshold of rowers, or in research on the impact of architecture and design on human physiology [181].
